# Therapeutic Potential of Glycosyl Flavonoids as Anti-Coronaviral Agents

**DOI:** 10.3390/ph14060546

**Published:** 2021-06-07

**Authors:** Patrícia I. C. Godinho, Raquel G. Soengas, Vera L. M. Silva

**Affiliations:** 1 LAQV-REQUIMTE, Department of Chemistry, University of Aveiro, 3810-193 Aveiro, Portugal; patricia.godinho@ua.pt; 2 Department of Organic and Inorganic Chemistry, University of Oviedo, Julián Clavería 7, 33006 Oviedo, Spain

**Keywords:** coronavirus disease, SARS-CoV-2, flavonoids, glycosyl flavonoids, antivirals, spike glycoprotein, protease 3CL^pro^

## Abstract

The COVID-19 pandemic, caused by *severe acute respiratory syndrome coronavirus 2* (SARS-CoV-2), has spread all over the world, creating a devastating socio-economic impact. Even though protective vaccines are starting to be administered, an effective antiviral agent for the prevention and treatment of COVID-19 is not available yet. Moreover, since new and deadly CoVs can emerge at any time with the potential of becoming pandemics, the development of therapeutic agents against potentially deadly CoVs is a research area of much current interest. In the search for anti-coronaviral drugs, researchers soon turned their heads towards glycosylated flavonoids. Glycosyl flavonoids, widespread in the plant kingdom, have received a lot of attention due to their widely recognized antioxidant, anti-inflammatory, neuroprotective, anticarcinogenic, antidiabetic, antimicrobial, and antiviral properties together with their capacity to modulate key cellular functions. The wide range of biological activities displayed by glycosyl flavonoids, along with their low toxicity, make them ideal candidates for drug development. In this review, we examine and discuss the up-to-date developments on glycosyl flavonoids as evidence-based natural sources of antivirals against coronaviruses and their potential role in the management of COVID-19.

## 1. Introduction

In December 2019, an outbreak of a severe pneumonia of unknown origin started in Wuhan, China. Soon after, similar cases were found in other countries around the world, and the number of infected people increased rapidly. At the end of January 2020, the World Health Organization (WHO) officially confirmed this pneumonia, caused by a new coronavirus, to be a public health emergency of global concern due to its rapid spread. The disease caused by this virus was named Coronavirus disease 2019 (COVID-19). On 11 March 2020, WHO declared it a global pandemic. Once the genetic analysis and information about the COVID-19 virus became available, the International Committee on Taxonomy of Viruses (ICTV) gave it its official name—*severe acute respiratory syndrome coronavirus 2* (SARS-CoV-2) [1,2]. Data received by WHO from national authorities of different countries by 28 January 2021 indicated that there were more than 98 million confirmed cases with COVID-19 and more than 2 million deaths. At the time of writing (12 April 2021), WHO reported a total of 135,057,587 confirmed cases of COVID-19 and 2,919,932 deaths worldwide (https://www.who.int/emergencies/diseases/novel-coronavirus-2019/situation-reports, accessed on 12 April 2021).

Even though protective vaccines are starting to be available, an effective antiviral agent for the prevention and treatment of COVID-19 is still lacking. Since new and deadly coronaviruses (CoVs) can emerge at any time with the potential of becoming pandemics, the development of therapeutic agents against potentially deadly CoVs is a research area of much current interest. In this regard, flavonoids were soon recognized as potential leads for the development of anti-coronaviral drugs.

Flavonoids are a large group of polyphenolic compounds consisting of different sub-classes, such as flavonols, flavones, flavanonols, flavanones, flavanols (or catechins), isoflavones, chalcones, and anthocyanidins [3]. In plants, flavonoids are formed as secondary metabolites that provide color to seeds, flowers, and fruits. Furthermore, they also provide protection by blocking the entry of solar ultraviolet (UV) radiation, and against pathogenic microorganisms; they also regulate plant growth and enzyme activity [4,5]. These compounds are widely distributed in the plant kingdom and are therefore the most common polyphenols in the human diet [6]. In fact, flavonoids have received a lot of attention due to their benefits on preventing and managing chronic diseases, such as diabetes, cancer, cardiovascular diseases, obesity, and much more [7,8]. These health benefits are mainly due to their highly recognized antioxidant [9,10], anti-inflammatory [11,12], neuroprotective [13,14], anticarcinogenic [15,16], antidiabetic [17], antimicrobial [18], and antiviral [19,20] properties, together with their capacity to modulate key cellular functions [21]. In view of the wide range of biological activities displayed by flavonoids, researchers soon turned their heads towards these natural products in the search for weapons against the new coronavirus.

In nature, flavonoids are extensively distributed in their glycosylated form, as glycosyl flavonoids. The glycosyl part of these hybrid derivatives is not only related to improved solubility and the absorption profile, but also has a great influence on recognition processes, and consequently, on the biological profile.

## 2. Severe Acute Respiratory Syndrome Coronavirus 2

**Figure 1:**
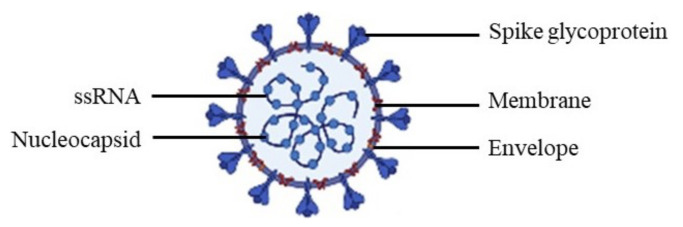
Schematic diagram of the coronavirus virion.

Coronaviruses (CoVs) can be found in humans and in different animal species such as bats, camels, and civets. They belong to the subfamily *coronavirinae* from the family *coronaviridae*. This subfamily can be divided into four genera, *Alphacoronavirus*, *Betacoronavirus*, *Gammacoronavirus*, and *Deltacoronavirus*. SARS-CoV-2 belongs to the *Betacoronavirus* family, which can only infect mammals [22,23]. CoVs are enveloped and spherical viruses that possess in their genome a single-stranded ribonucleic acid (ssRNA) that is 27–32 kilobases in length, with a positive polarity, which means that the base sequence orientation of the RNA is 5′→3′ [23]. The viral RNA contains open reading frames (ORF) that comprise two-thirds of the genome and consist of sixteen nonstructural proteins (nsp), which include the RNA-dependent RNA polymerase (RdRp) and other replicase proteins. These are primarily processed by the virally encoded 3C-like protease (3CL^pro^), with additional cleavage performed by the viral papain-like proteases (PL^pro^). The remaining one-third of the viral RNA encodes four essential structural proteins, such as the spike glycoprotein, envelope protein, membrane protein, and nucleocapsid protein (Figure 1) [24,25]. The genome of CoVs is one of the largest RNA genomes, giving extra plasticity to this family of viruses in accommodating and modifying genes [26]. In fact, CoVs have a high rate of mutation due to RdRp, which is responsible for the replication of RNA viruses, thus being more error-prone [23,27]. Until SARS-CoV-2 appeared, there were at least six known coronaviruses that could infect humans. The viruses that can cause severe acute respiratory syndromes are *severe acute respiratory syndrome coronavirus* (SARS-CoV), which caused an outbreak in 2002, *Middle East respiratory syndrome coronavirus* (MERS-CoV) that emerged in 2012, and SARS-CoV-2, causative of the current COVID-19 pandemic. The other four viruses, HCoV-NL63, HCoV-229E, HCoV-OC43, and HKU1, can cause mild upper respiratory diseases [23]. The genome of SARS-CoV-2 shows similarities to other *Betacoronavirus*, sharing ~80% sequence identity with SARS-CoV and ~50% sequence identity with MERS-CoV [28]. 

**Figure 2:**
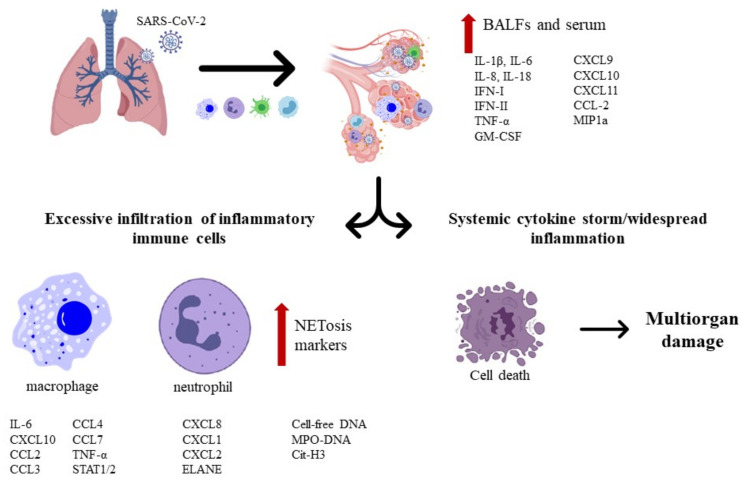
Overview of lung pathology in patients with *severe acute respiratory syndrome coronavirus 2.* The black arrows show the pathways of lung lesions after inhalation and entry of SARS-CoV-2 into the respiratory tract. The red arrows show the increase of inflammation markers.

The primary tropism of SARS-CoV-2 is the lungs since patients exhibit respiratory-like illnesses that progress to severe pneumonia. This virus enters the host via the respiratory tract, which first targets the airway and alveolar epithelial cells, vascular endothelial cells, and alveolar macrophages (Figure 2). In fact, these cells express receptors for SARS-CoV-2 entry, which reinforces the idea that they are the spot/point in early infection [28].

The CoVs are transmitted through respiratory droplets and direct contact with contaminated surfaces. The incubation period of SARS-CoV-2 is short, usually taking approximately five to six days, whereas SARS-CoV and MERS-CoV usually take around two to eleven days. The SARS-CoV-2 infection causes severe flu-like symptoms that can progress to acute respiratory distress, pneumonia, renal failure, and even death. The most common symptoms are fever, cough, dyspnea, fatigue, and muscle pain [1,28]. SARS-CoV-2 can also lead to myocardial injury, arrhythmic complications, and neurological complications such as headache, myalgia, anosmia, ageusia, and even stroke [29,30,31].

The SARS-CoV-2 infection in patients with severe symptoms lead to proinflammatory macrophages and neutrophils in bronchoalveolar lavage fluid (BALF), with elevated proinflammatory cytokines (IL-6 and IL-8, e.g.,) in the BALF, along with the high expression of inflammatory chemokines (CCL2, e.g.,) in macrophages (Figure 2).

A noticeable “cytokines storm” occurs in patients critically ill with COVID-19. These proinflammatory mediators can elevate C-reactive protein (CRP) from the liver through the signal transducer and activator of transcription 3 (STAT3)-IL-6 signaling, which can be correlated with the elevated production of serum IL-6. Additionally, patients with severe symptoms exhibit a pronounced formation of neutrophil extracellular traps (NETs) inside microvessels that are possible potentiators of pathogenesis. Dysfunction of pulmonary endothelial cells through vascular leakage and compromised barrier function can promote tissue edema and endotheliitis by the recruitment of activated neutrophiles and monocytes. Thus, this limits gas exchange and facilitates a hypoxic environment, leading to respiratory and organ failure [28]. 

Therefore, several strategies have been adopted to develop SARS-CoV-2 inhibitors since the first disease outbreak. The main strategies developed aim to block the attachment and entry of the virus into host cells and interfere with viral replication and translation in order to prevent the release of viruses and the infection of other cells. 

### 2.1. Spike Glycoprotein

The spike glycoprotein mediates the entry of SARS-CoV-2 into host cells. This glycoprotein assembles into stable homotrimers that protrude from the surface of mature virions and is critical for SARS-CoV-2 entry into the host cell [24]. The SARS-CoV-2 spike glycoprotein is a trimeric class I fusion protein consisting of two functional subunits: a receptor binding subunit (S1) and a membrane fusion (S2) [32]. The S1 subunit contains an N-terminal domain and a receptor-binding domain (RBD), which is responsible for binding to a host cell receptor. The S2 subunit contains basic elements, whose functions include fusing the membrane of the virus to the host cells [24]. The S1 subunit in CoVs has special domains that recognize different entry receptors. However, for SARS-CoV-2 to bind and enter the host cell, it must recognize the host angiotensin-converting enzyme 2 (ACE2) through the RBD. The main mutations in the RBD of the SARS-CoV-2 spike glycoprotein result in additional contacts with ACE2, explaining its higher affinity when compared to other CoVs [28,33]. The SARS-CoV-2 RBD includes two structural domains: the core and the external subdomains. The core subdomain is composed of five β strands arranged in an antiparallel manner and a conserved disulfide bond between two β strands. The external subdomain is dominated by a flexible loop that connects two β strands with a stabilized disulfide bond [34]. 

**Figure 3:**
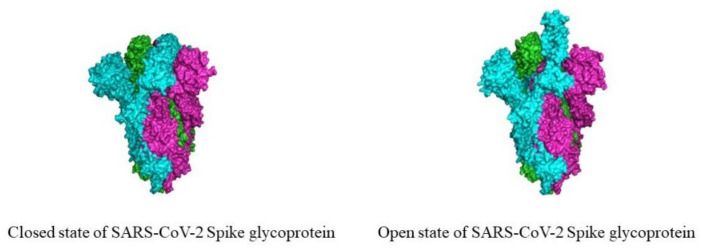
Cryo-EM structure of the SARS-CoV-2 spike glycoprotein. The closed state (**left**) and open state (**right**) of the spike glycoprotein (closed state PDB: 6VXX and open state PDB: 6VYB).

The spike glycoprotein has two conformational states that are referred to as the “down” conformation and the “up” conformation. The “down” conformation is the closed state in which the receptor is inaccessible to the fusion, whereas the “up” conformation is the open state, where the receptor is accessible (Figure 3). Thus, the fusion of SARS-CoV-2 and the host cell happens when the conformation is “up” [2,24]. The binding of SARS-CoV-2 and the host cell triggers a cascade of events that lead to the fusion of the cell and the viral membranes, which is required for cell entry. The spike glycoprotein exists in a metastable prefusion conformation, where the S1 and S2 subunits remain non-covalently bound. When the S1 subunit binds to the host cell receptor, it destabilizes the prefusion trimer, which leads the RBD of the S1 subunit to suffer conformational movements that transiently hide or expose the determinants of receptor binding, and to transition to the S2 subunit for a stable post-fusion conformation [2,24]. Both the S1 and S2 subunits of the spike glycoprotein are extensively decorated with N-linked glycans, possessing 22 N-linked glycan sites. Therefore, the surface of the envelope spike displays 66 N-linked glycosylation sites. These glycosylation sites mediate the protein folding, stability, and shape viral tropism. The spike glycoprotein is biosynthesized in the secretory pathway, where the nascent polypeptides are translocated into the endoplasmic reticulum lumen and are modified with the Glc_3_Man_9_GlcNAc_2_ glycan structure. This glycan undergoes rapid hydrolytic trimming, with the first steps involving the trimming of glucose units by α-glucosidases I and II. The intermediates from the truncated glycan are necessary for the recognition of calnexin chaperones and calreticulin that facilitate the protein folding. The correctly folded proteins with a Man_9_GlcNAc_2_ structure form Man_8_GlcNAc_2_ glycoproteins trimmed in the B branch that are translocated to the Golgi apparatus [32,35].

The receptor recognition mechanism of SARS-CoV-2 determines the infectivity, pathogenesis, and the host range of the virus [36]. In fact, several studies have shown that SARS-CoV-2 has higher affinity to ACE2 than the SARS-CoV, which has important implications on the potential animal-to-human transmission of SARS-CoV-2 [36,37]. The surface of ACE2 has two major binding hotspots that are essential for SARS-CoV-2 binding. The SARS-CoV-2 RBD interacts with ACE2 through hydrophilic residues located along the interface, which form a solid network of hydrogen bonds and salt bridge interactions [34]. The ACE2 possesses glycans on the interface; however, the glycosylation states differ on the tissue, cell type, and the age of the human host. Zhao et al. observed glycan-mediated interactions between the spike glycans and one ACE2 receptor glycan [38]. On the other hand, Lan et al. found that there are 13 hydrogen bonds and 2 salt bridges at the SARS-CoV-2 RBD-ACE2 interface. This hydrogen bonding interactions are made through tyrosine residues from the SARS-CoV-2 RBD with a polar hydroxy group on the ACE2. Furthermore, ACE2 has an N-acetyl-β-glucosamine (NAG) glycan attached to an asparagine residue. Although there was no evidence that the SARS-CoV-2 RBD interacts with the NAG glycan, this does not exclude the fact that the glycans after the first NAG may interact with the SARS-CoV-2 RBD. It has been proposed that the glycan–RBD interaction has an important role in the binding of SARS-CoV-2 to ACE2 [37]. 

### 2.2. 3C-Like Protease and Papain-Like Protease

Both 3C-like protease (3CL^pro^) and papain-like protease (PL^pro^) are non-structural proteins encoded by the SARS-CoV-2 genome on the ORF. After the fusion of SARS-CoV-2 with the host cell, the viral RNA is released into the cytosol, which is translated into the replicase proteins [15]. 3CL^pro^ and PL^pro^ cleave the replicase polyprotein into 15–16 non-structural proteins (nsps) at consensus cleavage sites [28,39,40,41]. Some of these nsps encode proteins with essential functions for virus-mediated RNA replication, so targeting these proteins is an effective antiviral strategy for suppressing viral genome replication in order to cure CoV infection. 

The main protease 3CL^pro^ of SARS-CoV-2 showed 96% sequence similarity to that of SARS-CoV, and the differences are only at twelve positions in the sequence alignment [42]. The number of amino acid residues in both proteases was identical (306), beginning from Ser1 to Gln306 [43]. Recent kinetic characterizations revealed only 2- and 3-fold differences in the kcat/Km values of SARS-CoV and SARS-CoV-2, 3CL^pro^ and PL^pro^, respectively [44]. However, recent studies also revealed marked differences in the kinetic values of SARS-CoV-2 PL^pro^ for ubiquitin (Ub) and interferon-stimulated gene product 15 (ISG15) as compared to its SARS-CoV counterpart [45]. The 3CL^pro^ is a dimeric cysteine protease where each monomer contains one independent active site, rendering the monomers less active than the dimer. The active site is composed of a cysteine and a histidine, where the cysteine acts as a nucleophile and the histidine residue acts as a base [39,40,41]. The two replicase polyproteins (pp1a and pp1ab), also known as promoters, are packed at almost a right angle [41]. These polyproteins contain three distinct subdomains, which are named domain I, domain II, and domain III. Domains I and II are antiparallel β-barrels with six strands and are responsible for the interaction with the substrate. Furthermore, these two domains are responsible for the autocatalytic ability of the cysteine and histidine residues since the active site is situated between these domains. Domain III consists of five α-helices and is attached to domain II through a long loop. This domain preserves the accurate conformation of the dimer and is therefore critical for the enzymatic activity. The removal of domain III results in the inactivation of the protease [39,41,46].

The substrate binds to the cleft that is located between domains I and II. The amino acids from the N terminus to the C terminus are numbered as -P4-P3-P2-P1↓P1′-P2′-P3′-P4′-, with the cleavage site between P1 and P1′ [41,47]. The 3CL^pro^ recognizes the residues from P4 to P1′, where P1, P2, and P1′ determine the specificity due to their high conservation. The amino acid glutamine in the P1 position is a fundamental requirement. The P2 position prefers leucine but can tolerate hydrophobic amino acids, whereas the P1′ position tolerates small residues like serine or alanine. The recognition beyond P1′ is not conserved [48]. Therefore, the active sites of 3CL^pro^ are composed of four subsites, namely S4, S2, S1, and S1′. These subsites are highly conserved among the CoVs 3CL^pro^, and since they are crucial for substrate recognition, they have been the subject of numerous drug design studies [49]. 

Great efforts have been made to target the SARS-CoV-2 3CL^pro^, while PL^pro^, which is also responsible for the processing of replicase proteins, has received much less attention. SARS-CoV PL^pro^ is also a cysteine protease, divided into four sub-domains: the N-terminal ubiquitin-like domain (Ubl, β1–3), the α-helical thumb domain (α2–7), the β-stranded finger domain (β4–7), and the palm domain (β8–1). In the finger sub-domain, four conserved cysteine (C189 and C192 on the loop between β4–5, C224 and C226 on the loop between β6–7) form a zinc finger belonging to the “zinc ribbon” fold group. The active site contains a classic catalytic triad, composed of Cys111, His272, and Asp286. Residue Cys111 is located 3.6 Å away from the catalytic histidine Hys272. Residue Hys272 donates a hydrogen bond to Asp286 with the length of 3.0 Å. The hydrogen bond between Asp108 and Trp93 (2.8 Å) strengthens the conformation of the oxygen anion hole [50].

Although the primary function of PL^pro^ is to process the viral polyprotein in a coordinated action with 3CL^pro^, this protease has the additional function of cleaving ubiquitin and ISG15 from host-cell proteins, thus allowing coronaviruses to escape the host innate immune responses [51]. SARS-CoV-2 PL^pro^ and SARS-CoV PL^pro^ are closely related and diverge from MERS-PL^pro^. In fact, SARS-CoV-2 and SARS-CoV PL^pro^ proteases share ∼82% amino acid sequence identity, so most of the structural features of the orthologs are conserved [52]. However, both proteases exhibit differences in their substrate preferences. Thus, SARS-CoV PL^pro^ strongly reduced the appearance of ubiquitinated substrates, with a lesser effect on ISGylated substrates, whereas SARS-CoV-2-PL^pro^ preferentially reduced the appearance of ISGylated protein substrates [53]. Considering the important roles of PL^pro^ in virus life cycle targeting, this protease is an attractive target for the development of antiviral drugs [54].

### 2.3. RNA-Dependent RNA Polymerase

RNA-dependent RNA polymerase (RdRp) is a core component of the virus replication and transcription complex (RTC), involved in the replication and transcription of the SARS-CoV-2 genome through the synthesis of a nascent RNA strand [55]. The RdRp possesses an active site with two magnesium ions that catalyze the phosphodiester bond formation—the RNA template and the ribonucleotide 5′-triphosphatases. Furthermore, there are two channels that meet in the active site, where the main channel contains the RNA template and the secondary channel allows the ribonucleotide units to build the RNA molecule in the 5′→3′ direction [56].

The RdRp of SARS-CoV-2 is composed of a core protein known as nsp12 [57] as well as two additional subunits, nsp8 and nsp7, required for proper activity [58]. The overall conformation of this RdRp has recently been reported [59] and is highly similar to the RdRp of SARS-CoV, sharing an amino acid identity of 96%. On the other hand, the homology between SARS-CoV-2 RdRp and MERS-CoV RdRp is only 70% [55]. The core protein is a single chain of approximately 900 amino acids and resembles a right hand, sub-divided into a finger domain, palm domain, and thumb domain [55,59]. Subunits nsp7 and nsp8 bind to the thumb, and an additional copy of nsp8 binds to the finger domain [55,59]. Two additional Zn ions are also required for the structural stability of the RdRp that are located outside the catalytic site. One of the Zn ion is attached to four amino acid residues (His295, Cys301, Cys306, and Cys310) in the N-terminal domain, while the second Zn ion is attached to four amino acid residues (Cys487, His642, Cys645, Cys646) located in the finger domain [55]. 

As RdRp is a crucial enzyme in the life cycle of coronaviruses, a huge number of attempts to develop anti-RdRp compounds are under clinical testing [60].

## 3. Structure and Function of Glycosyl Flavonoids

Glycosyl flavonoids are structurally composed of a flavonoid aglycone linked to a sugar moiety. Different sugar moieties can be found in its structure, such as glucose, galactose, rhamnose, arabinose, and rutinose [8]. Glycosylation has a tremendous impact on the biological properties of flavonoid derivatives [61]. Flavonoids are poorly soluble in aqueous solutions, in which they also have the tendency to for insoluble polymers. Upon glycosylation, the solubility of flavonoids in water is greatly enhanced, leading to an improvement of their pharmacological properties. Thus, glycosylation not only increases flavonoids bioavailability, but also decreases their acute toxicity or harmful effects [62]. The stability of flavonoids towards oxidative degradation is also affected upon glycosylation; thus, the addition of a sugar moiety can block the phenolic group, resulting in enhanced stability [63]. In general terms, most biological activities are usually less pronounced in glycosides, but some specific bioactivities, including the anti-*human immunodeficiency virus* (HIV) and anti-*rotavirus*, are enhanced [8].

Glycosyl flavonoids are divided into two groups: *O*-glycosyl flavonoids and *C*-glycosyl flavonoids. In nature, they exist primarily as *O*-glycosyl flavonoids, where the sugar moiety is linked by an *O*-glycosidic bond to the flavonoid aglycone. In the *C*-glycosyl flavonoids, the flavonoid and sugar group are linked by a *C*-glycosidic bond. The types of glycosidic bonds, and the regioselectivity and stereoselectivity of the glycosylation are related with the glycosyl transferase involved in their biosynthesis [61]. Despite *O*-glycosyl flavonoids being the most common and structurally diverse metabolites in plants, *C*-glycosyl flavonoids exhibit different activities and properties in comparison to the *O*-glycosyl flavonoids. Since *C*-glycosidic bonds are less prone to hydrolysis than *O*-glycosidic bonds, *C*-glycosyl flavonoids present enhanced stability towards enzymatic and chemical hydrolysis [6]. In fact, the deglycosylation of *C*-glycosyl flavonoids is not imperative for their absorption, and this is corroborated by the presence of intact *C*-glycosyl flavonoids in human urine after oral consumption [6]. 

### 3.1. Biological Activity of Glycosyl Flavonoids

Flavonoids have long been associated with their powerful antioxidant activities. Since oxidative stress has recently been proposed as an essential factor that increases the severity of COVID-19, interest in natural flavonoids has grown exponentially over the past year [64].

**Figure 4:**
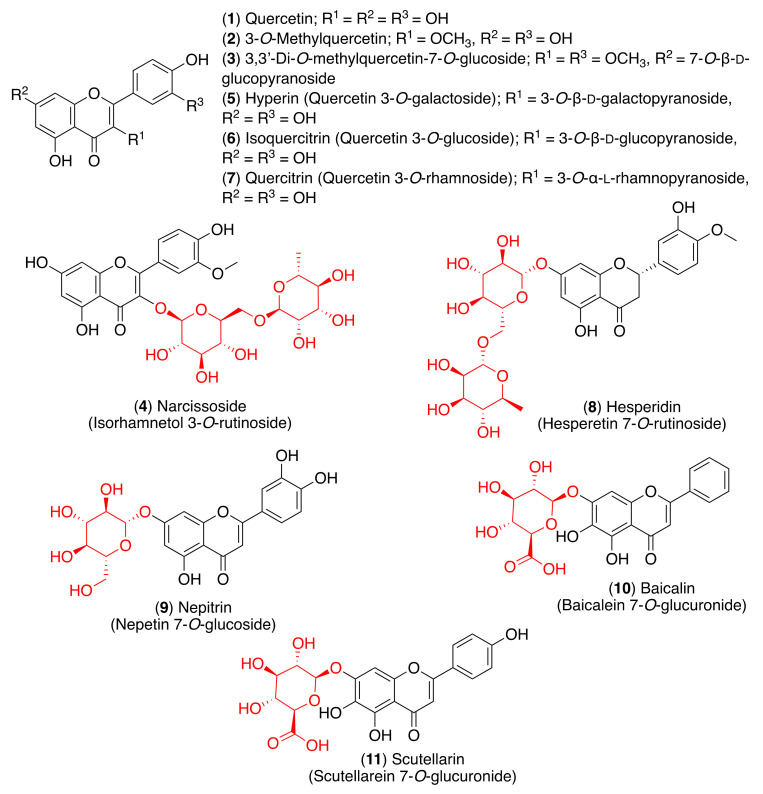
Anti-inflammatory and antioxidant glycosyl flavonoids.

As stated above, flavonoids can be found in nature in their glycosidic form and the sugar molecules attached to the flavonoid aglycone play a crucial role in biological activity. In this regard, glycosylation seems to generally reduce antioxidant activity. For example, the radical scavenging activities of quercetin and its glycosides, isolated from *Halimodendron halodendron*, were determined in the DPPH assay, and quercetin (**1**) and 3-*O*-methylquercetin (**2**) presented the highest antioxidant activities. The 3,3′-di-*O*-methylquercetin-7-*O*-glucoside (**3**) and narcissoside (**4**) presented weak radical scavenging activity (Figure 4) [65]. Choi et al. tested the in vitro and cellular antioxidant capacities of quercetin and its glycosides. Although isoquercitrin (**6**) and quercitrin (**7**) showed moderate antioxidant activity, quercetin (**1**) and hyperin (**5**) displayed strong cellular antioxidant capacity (Figure 4) [66]. Hesperidin (**8**) showed high antioxidant activity; however, it has been shown that this property is not limited to radical scavenging. In fact, hesperidin has the capability to attenuate tissue damage through antioxidant cellular defenses via the ERK/Nrf2 signaling pathway, which leads to the decrease in intracellular pro-oxidants and an increase in bilirubin as an internal antioxidant [67].

Besides their antioxidant activity, flavonoids are also well-known anti-inflammatory agents due to their cytokine-modulatory effects. The severity of COVID-19 infection is related to hypercytokinemia, an exaggerated immune response associated with an excessive and uncontrolled release of proinflammatory cytokine mediators—the so-called cytokine storm [68]. Choi et al. also tested the anti-inflammatory activity of quercetin (**1**) and its glycosides [66]. Although every compound presented anti-inflammatory activity, quercetin showed the highest efficacy in suppressing nitric oxide (NO) production, decreasing inducible nitric oxide synthase (iNOS) and cyclooxygenase-2 (COX-2) expression, and suppressing nuclear factor kappa B (NF-κB) activation. Quercetin (**1**) and quercitrin (**7**) showed a dose-dependent decrease of iNOS levels, while treatment with hyperin (**5**) and isoquercitrin (**6**) presented a non-dose-dependent suppressive effect. Consequently, the other mediator of the pro-inflammatory process, COX-2, was also attenuated in a dose-dependent manner. These results indicated that quercetin and its glycosides inhibit NO production in LPS-stimulated RWAS 264.7 cells through the attenuation of iNOS and COX-2 expression. The decrease of these levels is induced through the suppression of NF-κB activation via phosphorylation, since NF-κB upregulates the iNOS and COX-2 expression [66]. Hesperidin (**8**) also showed anti-inflammatory activity through down-regulation of iNOS and COX-2 in various in vitro and in vivo studies [66]. More recently, hesperidin was found to attenuate high levels of angiotensin II (AngII) in hypertensive rat models [69]. The anti-inflammatory effect mediated through anti-angiotensin action has previously been described for other flavonoid derivatives, such as glycosyl flavonoid nepitrin (**9**) [70].

Several other glycosyl flavonoids have been reported to modulate inflammatory mediators or signaling cascades, including toll-like receptors (TLRs) and NLR family pyrin domain containing 3 (NLRP3) inflammasomes. For example, baicalin (**10**) showed an effective protection of neurons from microglia-mediated neuroinflammation via the suppression of NLRP3 inflammasomes and the TLR4/NF-κB signaling pathway [71], while scutellarin (**11**) displayed the suppression of NLRP3 inflammasome activation in macrophages [72]. Considering that the deregulation of TLRs and NLRP3 is closely related to the severity of SARS-CoV-2 pathology, it can be assumed that glycosyl flavonoids could exert significant antiviral and immunomodulatory effects mediated through TLRs or NLRP3 inflammasomes in COVID-19 patients. However, these potential effects need to be evaluated further in well-defined pre-clinical and clinical studies.

**Figure 5:**
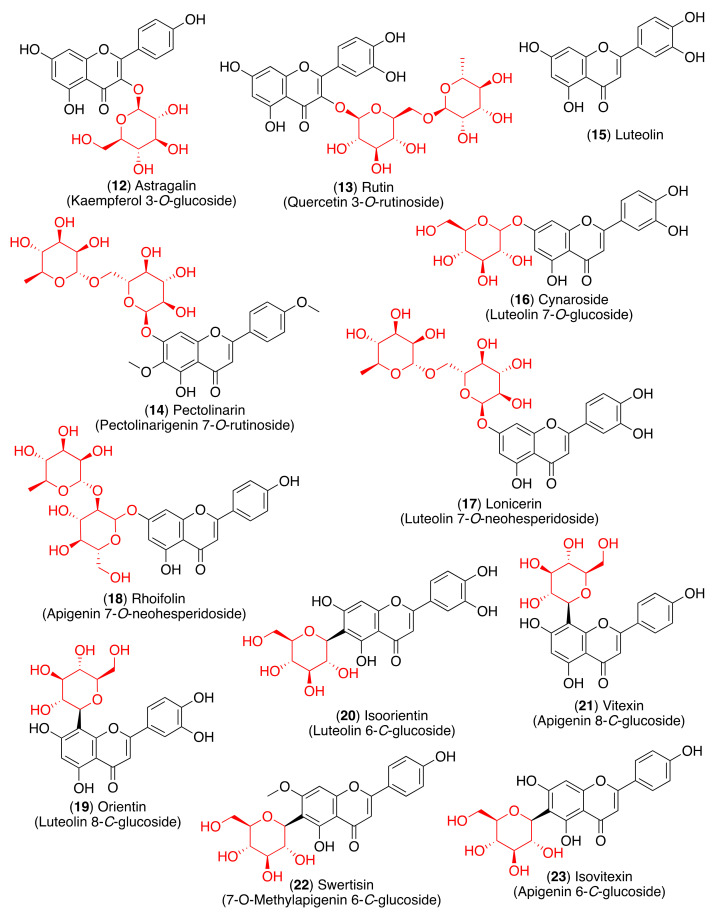
Glycosyl flavonoids with α-glucosidase inhibitory activity.

As most viral envelope glycoproteins contain N-linked glycans, α-glucosidase inhibitors have been proposed as potentially useful broad-spectrum antiviral agents based on their activity on a variety of enveloped viruses [73]. In this regard, some flavonoids and glycosyl flavonoids have been evaluated for their inhibitory activity of glucosidases. Hyperin (**5**), quercitrin (**7**), baicalin (**10**), astragalin (**12**), rutin (**13**) and pectolinarin (**14**) (Figure 4 and Figure 5) exhibited low α-glucosidase inhibitory activity. On the other hand, luteolin (**15**) and cynaroside (**16**) showed strong inhibitory activity against α-glucosidase (IC_50_ 1.7–3.5 µM), while lonicerin (**17**) and rhoifolin (**18**) inhibited α-amylase strongly (IC_50_ 8.4–84.0 µM) [74]. Shibano et al. studied the antioxidant and antidiabetic activity of several glycosyl flavonoids. The DPPH radical scavenging activities of the glycosyl flavonoids isolated from *Commelina communis* L., showed that isoquercitrin (**6**), cynaroside (**16**), orientin (**19**), and isoorientin (**20**) (Figure 5) were potent antioxidants. In addition to their antioxidant activity, narcissoside (**4**), isoquercitrin (**6**), vitexin (**21**), and swertisin (**22**) (Figure 4 and Figure 5) inhibited α-glucosidase (IC_50_ 0.51, 0.24, 0.42 and 0.37 µM, respectively) [75]. Choo et al. reported the α-glucosidase inhibitory potential of vitexin (**21**) and isovitexin (**23**) (Figure 5) isolated from the leaves of *Ficus deltoidea*. In this in vivo study, vitexin (**21**) and isovitexin (**23**) showed a high percentage of postprandial blood glucose reduction on sucrose-loaded normoglycemic mice and induced diabetic rats. Therefore, the report supports the use of vitexin and isovitexin for managing diabetes mellitus and its complications [76].

### 3.2. Antiviral Activity of Glycosyl Flavonoids

The antiviral properties of flavonoids were discovered in the 1940s [77,78,79]. Since then, flavonoids have been studied as possible therapeutic agents against many viruses. In fact, they can work through several different mechanisms, such as blocking the attachment and entry of the virus into host cells, interfering with various stages of viral replication processes or translation, and polyprotein processing to prevent the release of the viruses to infect other cells [80]. 

**Figure 6:**
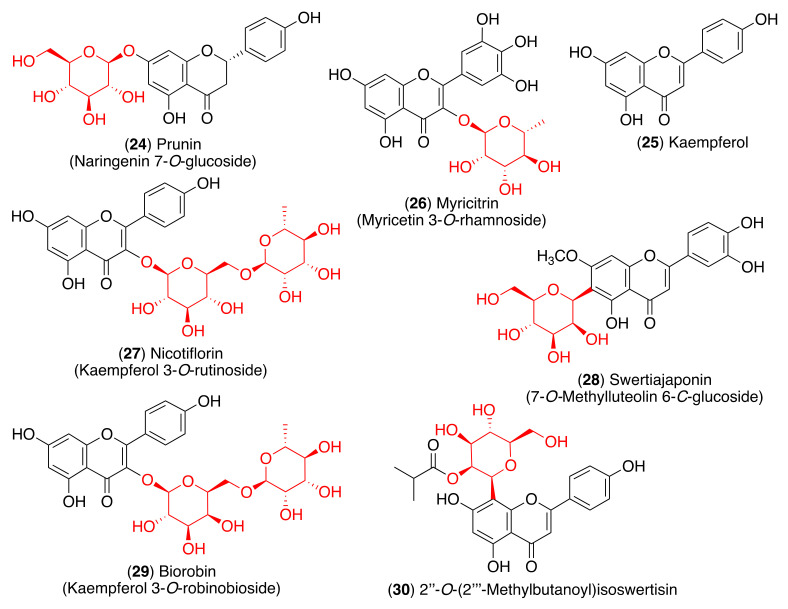
Antiviral glycosyl flavonoids.

Since glycosyl flavonoids are widely distributed in the plant kingdom, they have also been studied as antiviral agents. Indeed, several glycosyl flavonoids have demonstrated inhibitory effects against several viruses (Figure 6, Table 1) [81,82,83,84,85,86,87,88,89,90,91,92,93,94,95].

For example, isoquercitrin (**6**) has the ability to protect mice from the *Ebola virus* when given prior to infection. Even though the mechanism of action is unknown, isoquercitrin targets the early steps of viral entry (IC_50_ 5.3 µM) [81]. Another quercetin glucoside, quercitrin (**7**), was found to inhibit the initial stage of virus replication in the *influenza A virus* infection [82]. Moreover, glycosyl flavonoid rutin (**13**) inhibits the *Enterovirus A71* 3C protease with an IC_50_ of 109.6 ± 1.1 µM, significantly reducing viral plaque formation and the cytopathic effect [83]. This inhibition is associated with the suppression of the MERK-ERK signaling pathway that is required for an efficient *Enterovirus A71* replication [84]. Baicalin (**10**) is another glycosyl flavonoid known to be an antiviral agent against *human immunodeficiency virus type 1* (HIV-1), *influenza A virus,* and *Enterovirus A71* (Table 1). Baicalin can inhibit the entry of HIV-1 since it interferes with the interaction of the envelope proteins of the virus with the host immune cells [85].

Baicalin can increase the interferon-gamma (INF-γ) in human CD4+ and CD8+T lymphocytes (CTL) and natural killer cells (NK) during an *influenza A virus* infection. The induction of INF-γ leads to the activation of Janus Kinase/Signal Transducer and Activator of Transcription (JAK/STAT-1) signaling pathway, leading to the expression and secretion of INF-γ [86]. In addition, baicalin exhibits potent antiviral activity for *Enterovirus A71*. Regarding the mode of action, baicalin blocked the expression of mRNA and polymerase in the early stages of infection by decreasing the expressions of Fas ligand (FasL) and caspase-3 that inhibit the *Enterovirus A71* apoptosis in Rhabdomyosarcoma cells. Moreover, baicalin suppresses the NF-κB signaling pathway, decreasing the secretion of cytokines [87].

## 4. Glycosyl Flavonoids as Anti-Coronaviral Agents 

The COVID-19 epidemic caused by the novel coronavirus (SARS-CoV-2) infection is a public health emergency of international concern. Despite several vaccines having been approved, the infection is still spreading at an alarming rate. In the absence of confirmed effective treatments and due to the public health emergency, it became crucial to study the possible effects of natural products for the management of SARS-CoV-2 [96]. Since the outbreak of SARS-CoV-2 in China, patients have been treated with traditional Chinese medicine as a first-line drug [97]. In February 2020, the rate of this treatment was at 87%, with only 5% of patients showing the worst clinical signs. The Qingfei Paidu Decoction, a formula consisting of 21 components including both herbs and mineral drugs, showed an effectiveness of 92% in patients at all stages of infection. Therefore, Yang et al. identified 129 constituents clustered into 14 groups, from which 45% were flavonoids [98]. These findings sparked interest in the study of flavonoids and glycosyl flavonoids as potential anti-SARS-CoV-2 agents [99,100]. In addition, several flavonoids have been identified to inhibit other CoVs, such as SARS and MERS.

The anti-coronaviral action of glycosyl flavonoids is in part due to the inhibition of the enzymatic activity of key targets involved in processes of virus replication, such as SARS-CoV-2 3CL^pro^, spike glycoprotein, SARS-CoV-2 PL^pro^, and RdRp. On the other hand, during viral infection, changes in the body’s antioxidant defense system lead to oxidative stress, which contributes to viral pathogenesis by stimulating inflammation, loss of immune function, and increased viral replication that may occur due to the activation of the nuclear factor kappa B (NF-*κ*B) transcription pathway and may lead to a cytokine storm. The significant antioxidant action of flavonoids contributes to the reduction of reactive oxygen species (ROS) accumulation in the body, which might contribute to retard coronavirus-activated apoptotic signaling. Thus, the mechanism of oxidative stress can be a key mechanism for controlling inflammatory processes arising from the virus action [101].

The 3CL^pro^ is involved in the replication and transcription of the viral RNA in the host cells. Since SARS-CoV and MERS-CoV proteases are very similar to the SARS-CoV-2 protease, inhibitors of the first two viruses are expected to inhibit the replication and transcription of the genomic RNA of SARS-CoV-2. Flavonoid quercetin (**1**) showed good inhibition of SARS-CoV 3CL^pro^ with IC_50_ values of 73 ± 4 µM [102]. The quercetin glycoside isoquercitrin (**6**) was found to block the enzymatic activity of MERS-CoV 3CL^pro^. The docking study showed that the glucosyl moiety binds strongly to the S1 subsite of MERS-CoV 3CL^pro^ through a hydrogen bond [103]. Jo et al. found that pectolinarin (**14**) and rhoifolin (**18**) have an inhibitory activity towards SARS-CoV 3CL^pro^ with IC_50_ values of 37.78 and 27.45 µM, respectively (Table 2). The high binding affinity to the S1 and S2 subsites of SARS-CoV 3CL^pro^ is associated with the presence of sugar moieties [104].

Su et al. described the anti-SARS-CoV-2 potential of the Shuanghuanglian preparation, a Chinese traditional patent medicine with a long history of treating respiratory tract infections in China, and identified glycosyl flavonoid baicalin (**10**) as the major bioactive ingredient [105]. Baicalin was reported as an inhibitor of SARS-CoV-2 3CL^pro^ through an enzymatic assay in combination with the ITC, ESI-MS, and X-ray protein crystallography. Although several other small molecules have been declared 3CL^pro^ inhibitors [106,107,108,109], this was the first report in which the binding with 3CL^pro^ was validated by ITC and complex structure. Thus, baicalin was tested by the FRET-based protease assay, showing an IC_50_ of 6.41 μM against SARS-CoV-2 3CL^pro^ (Table 2). To validate the binding of baicalin with 3CL^pro^ and exclude the possibility of the pan-assay interference compounds, their binding affinities with the protease were measured by ITC. The resulting K_d_ of baicalin and baicalein binding with SARS-CoV-2 3CL^pro^ was 11.50 μM. The good correlation with the IC_50_ value demonstrated that the specific binding of baicalin with the enzyme was responsible for the antiviral activity. The antiviral efficacy of baicalin was further evaluated against a clinical isolate of SARS-CoV-2 in Vero E6 cells, showing dose-dependent inhibition of SARS-CoV-2 replication (EC_50_ = 27.87 μM). In a further study, both baicalin (**10**) and pectolinarin (**14**) revealed prominent inhibitory activity against SARS-CoV-2 3CL^pro^, with measured IC_50_ values of 34.71 and 51.64 µM, respectively (Table 2) [110]. To deduce the binding mode and binding affinity, Jo et al. performed an in silico docking study which showed fundamental differences in the binding of baicalin (**10**) as compared to pectolinarin (**14**). In pectolinarin, the sugar moiety occupies the S1 and S2 subsites of 3CL^pro^, whereas the 4*H*-chromen-4-one moiety is in the S2 and S’3 subsites. In baicalin, the binding mode is severely influenced by the presence of the glucuronate sugar moiety. Thus, the important hydrogen bonds with Glu166 are formed by the 6-hydroxy group linked to the chromen-4-one moiety and also by the 5-hydroxy group attached to the glucuronate moiety.

These findings prompted many studies on the inhibition of 3CL^pro^ by glycosyl flavonoids using molecular docking technology and simulation studies (Table 3). For example, a computational study showed that narcissoside (**4**), a flavonoid present in several wild plants, fits perfectly in the active site of 6W63, the experimental structure of SARS-CoV-2 3CL^pro^ [111]. Narcissoside (**4**) displayed an increased inhibitory potency compared to the standard inhibitor **X77** [(*R*)-*N*-(4-*tert*-butylphenyl)-*N*-(2-(cyclohexylamino)-2-oxo-1-(pyridin-3-yl)ethyl)-1*H*-imidazole-5-carboxamide], as it showed thirteen hydrogen bonds with nine amino acids in the active site pocket of 6W63, while X77 showed only four hydrogen bonds with four amino acids (Table 3).

Recently, quercetin (**1**) was found to have the ability to inhibit the 3CL^pro^ of SARS-CoV-2 in an experimental in vitro molecular screening [112]. Despite being a potent in vitro inhibitor of 3CL^pro^, low in vivo bioavailability of quercetin hampers its potential as a therapeutic anti-coronaviral agent. In order to address this issue, Rizzuti et al. investigated the effect of glycosylation on quercetin binding to 3CL^pro^ by using rutin (**13**), a natural glycosylated conjugate of quercetin, as a model. Combining experimental (spectroscopy and calorimetry) and simulation techniques (docking and molecular dynamics simulations), it was found that the sugar adduct does not hamper rutin binding to 3CL^pro^, and the conjugated compound preserves a high potency (inhibition constant in the low micromolar range, K_i_ = 11 μM). Such validation constitutes an important proof-of-concept that the presence of a sugar adduct allows the glycoside form to retain the key bioactive features of the aglycone lead compound [113].

**Figure 7:**
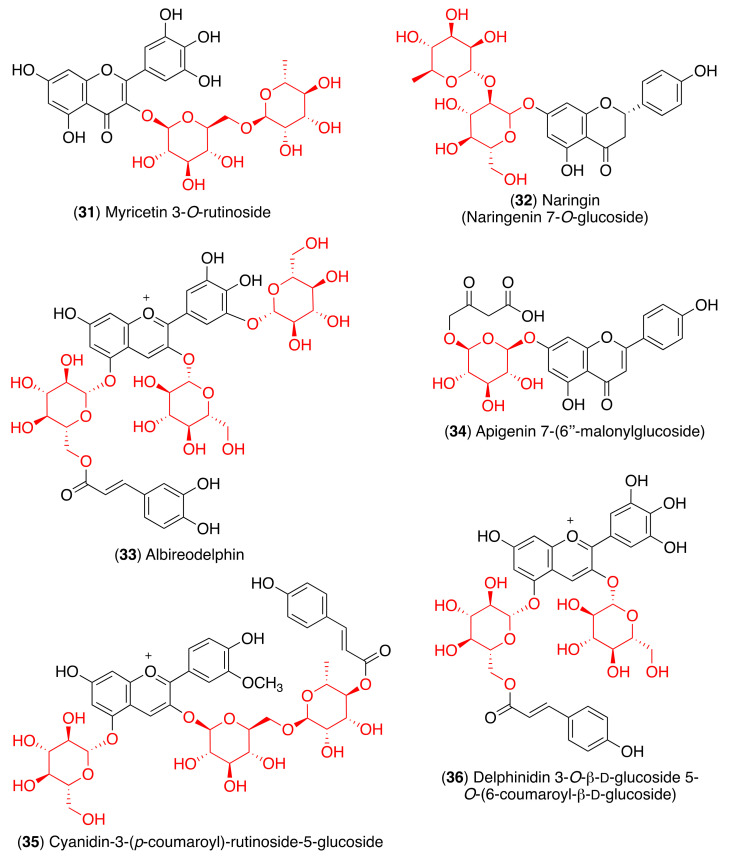
Anti-coronaviral glycosyl flavonoids.

However, another molecular docking and simulation study showed that quercitrin (**7**), myricetin 3-*O*-rutinoside (**31**), and rutin (**13**) are potential drug candidates with high affinity to the active pocket of SARS-CoV-2 3CL^pro^ [114] (Figure 7). The 3CL^pro^ inhibitory activity of rutin (**13**) was also confirmed in later studies [115,116]. Thus, a molecular docking approach demonstrated high affinity of rutin (**13**), nicotiflorin (**27**), and their human metabolites to SARS-CoV-2 3CL^pro^ and also to RdRp [115]. Additionally, Agrawal et al. described that rutin matched very well with the 6GLU7 binding pocket of 3CL^pro^ and that it is able to form several hydrogen bonds and σ-π stacking interactions with various amino acids, suggesting that it may be a good inhibitor [116]. Furthermore, a docking study revealed scutellarin (**11**) as a potent candidate for targeting 3CL^pro^ [117].

In contrast, the spike glycoprotein plays a crucial role in SARS-CoV-2 infection since it is involved in viral attachment, fusion, and entry into host cells, thus promoting its pathogenesis. This glycoprotein binds to the ACE2 receptor through hydrogen bonds and salt bridges in order to enter the host cell. Therefore, SARS-CoV-2 spike glycoprotein is a potential therapeutic target, which has prompted many efforts to study the binding ability of natural compounds to the functional domains of this protein by means of molecular docking (Table 3). These computational studies provide valuable information for the discovery of the potential inhibitors of SARS-CoV-2; however, they have disadvantages such as low accuracy and high rate of false positive results. Despite these drawbacks, the biological evaluation of the potential anti-coronaviral agents identified through molecular docking can be useful, not only for the identification of novel pharmacological leads, but also for the improvement of screening accuracy. 

Pandley et al. reported that according to the results obtained using molecular docking, flavonoid quercetin (**1**) binds to the spike glycoprotein with a higher binding affinity than hydroxychloroquine [118], an analog of chloroquine that has received a lot of attention since it emerged as a potential therapeutic option against SARS-CoV-2 [119]. The same authors reported that glycosyl flavonoid baicalin (**10**) shows a very high affinity to spike glycoprotein, not only higher than standard drugs like hydroxychloroquine and abacavir, but also higher than quercetin (Table 3) [120]. Another in silico study that points to glycosyl flavonoids as effective antiviral agents was recently disclosed [121]. This report showed that glycosyl flavonoid naringin (**32**), widespread in citrus fruits, presented a high affinity to the SARS-CoV-2 spike glycoprotein. These computational studies suggest that glycosyl flavonoids and spike glycoprotein could create stable complexes. The in silico results have already been confirmed for several flavonoid derivatives. Thus, Biagioli et al. recently reported molecular docking studies suggesting that anthocyanidin pelargonidin binds a fatty acid binding pocket to the receptor binding domain of the SARS-CoV2 spike glycoprotein. In vitro studies subsequently demonstrated that pelargonidin significantly reduces the binding of the SARS-CoV2 spike glycoprotein to ACE2, affecting the virus uptake and replication [122]. In a recent report about the inhibition effect of Anatolian propolis against SARS-CoV-2, in silico studies showed that several flavonoids bind stronger to the SARS-CoV-2 spike glycoprotein than the reference molecule, hydroxychloroquine. Then, the ability of these flavonoids to inhibit the interaction of the SARS CoV-2 S1 spike glycoprotein and ACE-2 was tested in vitro. Hesperidin aglycone hesperetin was the best inhibitor against the SARS CoV-2 S1 spike glycoprotein and ACE-2, with an IC_50_ value of 16.88 mM [123].

In order to identify the multiple target binding potential, Rameshkumar et al. screened a library of flavonoid compounds in silico against the two major targets of SARS-CoV-2—3CL^pro^ and spike glycoprotein—as well as with the RdRp protein target [124]. The docking studies revealed that glycosyl flavonoids albireodelphin (**33**), apigenin 7-(6″-malonylglucoside) (**34**), cyanidin-3-(*p*-coumaroyl)-rutinoside-5-glucoside (**35**), and delphinidin 3-*O*-β-d-glucoside 5-*O*-(6-coumaroyl-β-d-glucoside) (**36**) had high binding energy values against all the three protein targets studied. In addition, a drug-likeness analysis reveals that these glycosyl flavonoids are non-carcinogenic and non-toxic, suggesting that these phytocompounds can be potential therapeutic agents against SARS-CoV-2.

Hiremath et al. studied in silico the ability of the phytochemicals present in *Phyllanthus amarus* and *Andrographis paniculata* to inhibit SARS-CoV-2 target proteins, including 3CL^pro^ and the spike glycoprotein, as well as RdRp and PL^pro^ [125]. In this report, the glycosyl flavonoid isoquercitrin (**6**) showed good binding affinities with all the four SARS-CoV-2 target proteins. This suggests that isoquercitrin, which demonstrated multiple target binding abilities in the docking analysis and a higher dock score than remdesivir, can be a potential candidate for fighting SARS-CoV-2. However, in vitro evaluation is needed to authenticate the prediction studies.

## 5. Conclusions

The SARS-CoV-2 outbreak has become a threat to the global population and health care systems. This virus has infected many people due to its quick spread, leading to the collapse of health care systems. In fact, the SARS-CoV-2 infection can cause respiratory and organ failure, which ultimately result in the death of infected patients. Despite the approval of several vaccines against COVID-19 infection, there is no defined curative treatment for SARS-CoV-2 or any related human coronavirus infection, such as SARS-CoV and MERS-CoV. Hence, the development of antiviral drugs against SARS-CoV-2 is considered urgent, not only to fight COVID-19, but also to be prepared for the very possible appearance of other human pathogenic coronaviruses.

In this review, we have discussed the information reported in the literature about glycosyl flavonoids as potential antiviral drugs for the inhibition of SARS-CoV-2 infection. Glycosyl flavonoids, structurally characterized by a flavonoid aglycone linked to a sugar moiety, are widely distributed in the plant kingdom. These natural products are highly recognized for their capacity to regulate antiviral, anti-inflammatory, and immunomodulatory responses, which also denotes their potential importance in the treatment of COVID-19. Recent reports also suggest that glycosyl flavonoids are promising inhibitors of SARS-CoV-2 target proteins (3CL^pro^, spike glycoprotein, PL^pro^, and RdRp), which would inhibit viral entrance in the cell, replication, and transcription. Despite the promising results of the studies published thus far on the use of glycosyl flavonoids against COVID-19, further studies focused on pharmacological in vitro and in vivo assays are needed to evaluate the applicability of these derivatives in the development of treatments against SARS-CoV-2. In addition, more glycosyl flavonoid analogues are required, especially those designed based on structure–activity relationship studies. In this sense, *C*-glycosyl flavonoids are especially important because, despite being naturally less abundant and having been much less studied than *O*-glycosyl flavonoids, they are remarkably more stable.

In this review, we believe that we have painted an accurate picture of the current status of glycosyl flavonoids as therapeutic substances targeting COVID-19, and we hope that this will provide a good starting point for researchers. 

## Figures and Tables

**Table 1:** Antiviral activity of glycosyl flavonoids.

Virus	Compound	IC_50_ (μM)	Reference
*Ebola virus*	Isoquercitrin (**6**)	5.3	[81]
*Enterovirus A71*	Baicalin (**10**)	11.1	[87]
	Hesperidin (**8**)	- *^a^*	[88]
	Prunin (**24**)	- *^a^*	[89]
	Rutin (**13**)	109.6	[83]
*Hepatitis B virus*	Isovitexin (**23**)	- *^a^*	[90]
*Herpes simplex virus*	Rutin (**13**)	- *^a^*	[91]
	Nicotiflorin (**27**)	- *^a^*	[91]
	Biorobin (**29**)	- *^a^*	[91]
*Human immunodeficiency virus type 1*	Baicalin (**10**)	- *^a^*	[85]
	Myricitrin (**26**)	7.9	[92]
*Influenza A virus*	2″-*O*-(2‴-Methylbutanoyl) isoswertisin (**30**)	147.8	[93]
	Quercitrin (**7**)	- *^a^*	[82]
	Baicalin (**10**)	- *^a^*	[86,94]
*Respiratory syncytial virus*	Isoorientin (**20**)	12.7	[95]
	Swertisin (**22**)	112.0	[95]
	Isovitexin (**23**)	23.1	[95]
	Swertiajaponin (**28**)	27.1	[95]

*^a^* Not described.

**Table 2:** Anti-coronaviral activity of glycosyl flavonoids.

Target	Compound	IC_50_ (μM)	Reference
MERS-CoV 3CL^pro^	Isoquercitrin (**6**)	- *^a^*	[103]
SARS-CoV 3CL^pro^	Pectolinarin (**14**)	37.78	[104]
	Rhoifolin (**18**)	27.45	[104]
SARS-CoV-2 3CL^pro^	Baicalin (**10**)	6.41 34.71	[105] [110]
	Pectolinarin (**14**)	51.64	[110]

*^a^* Not described.

**Table 3:** Molecular docking analysis of anti-coronaviral glycosyl flavonoids.

Target	Compound	Amino Acid Residues	Reference
SARS-CoV-2 3CL^pro^	Narcissoside (**4**)	Arg188, Glu166, His164, Cys145, Asn14, Cys44, His41, Gln192, Thr190	[111]
	Rutin (**13**)	Leu141, Thr26, Cys145, His41, Met49, Tyr54, Met165, Glu166	[113,114,115,116]
	Quercitrin (**7**)	Thr26, Phe140, Leu141, Gly143, His163, Arg188, Met49, Cys145	[114]
	Nicotiflorin (**27**)	Met49, Met165, Glu166, Thr190, Cys145, His41	[115]
	Myricetin 3-*O*-rutinoside (**31**)	Tyr54, His41, Met49, Met165, Thr26, Cys145, Ser144, Leu141, Gly143, Asn142, His163, Glu166	[114]
	Albireodelphin (**33**)	Lys5, Val125, Lys137, Ser139, Thr199, Glu288	[124]
	Apigenin 7-(6″-malonylglucoside) (**34**)	Gln110, Thr111, Thr292, Asp295	[124]
	Cyanidin-3-(*p*-coumaroyl)-rutinoside-5-glucoside (**35**)	Thr111, Gln110, Asn151	[124]
	Delphinidin 3-*O*-β-d-glucoside 5-*O*-(6-coumaroyl-β-d-glucoside) (**36**)	Asp153, Asn151, Ser158, Thr111, Ile249	[124]
	Isoquercitrin (**6**)	Leu141, His163, Met165	[125]
Spike glycoprotein	Baicalin (**10**)	Lys964, Gln965, Leu962, Thr961, Ser1003, Ala958, Tyr1007, Gln1011, Gln1010, Agr1014	[120]
	Naringin (**32**)	Asn290, Ile291, His374, Leu370, Leu410, Ala413, Pro415, Phe438, Gln442, Asp367, Thr371, Lys441, Glu406, Ser409	[121]
	Albireodelphin (**33**)	Lys621, Asp623, Phe793, Lys798, Asp760, Asp761, Trp800, Glu811, Cys813, Ser814	[124]
	Apigenin 7-(6″-malonylglucoside) (**34**)	Asp452, Trp617, Tyr619, Lys621, Asp760, Asp761, Trp800	[124]
	Cyanidin-3-(*p*-coumaroyl)-rutinoside-5-glucoside (**35**)	Asp164, Ile548, Ser549, Arg553, Arg555, Asp760, Asp761	[124]
	Delphinidin 3-*O*-β-d-glucoside 5-*O*-(6-coumaroyl-β-d-glucoside) (**36**)	Ser549, Arg553, Arg555, Thr556, Cys622, Asp623, Asp760, Asp761	[124]
	Isoquercitrin (**6**)	Thr998, Arg995, Asp994	[125]
RdRp	Rutin (**13**)	Tyr455, Arg553, Ala554, Asp452, Arg624, Asp623, Asn691, Ser759, Thr556, Asp760, Cys622	[115]
	Albireodelphin (**33**)	Arg348, Asp350, His378, Asp382, Phe390, Asn394, Asn397, Glu398, His401, Glu402, Arg514	[124]
	Apigenin 7-(6″-malonylglucoside) (**34**)	Als348, Asp350, His378, Asp382, Gly395, Asn394, His401	[124]
	Cyanidin-3-(*p*-coumaroyl)-rutinoside-5-glucoside (**35**)	Ser44, Asp206, Als348, Asp350, Asn397, Glu398, Ser511, Arg514	[124]
	Delphinidin 3-*O*-β-d-glucoside 5-*O*-(6-coumaroyl-β-d-glucoside) (**36**)	Try127, His345, Ala348, Asp350, Asp382, Tyr385, Asn394, Asn397, Arg401, His505, Arg514, Tyr515	[124]
	Isoquercitrin (**6**)	Ala125, His133	[125]
SARS-CoV-2 PL^pro^	Isoquercitrin (**6**)	His74, Arg83, Tyr155, Asn157, His176	[125]

## Data Availability

No new data were created or analyzed in this study. Data sharing is not applicable to this article.
